# Effective dosing of L-carnitine in the secondary prevention of cardiovascular disease: a systematic review and meta-analysis

**DOI:** 10.1186/1471-2261-14-88

**Published:** 2014-07-21

**Authors:** Ruiping Shang, Zhiqi Sun, Hui Li

**Affiliations:** 1 Department of Cardiology, Daqing General Hospital Group, Oilfield General Hospital, No. 9 Zhongkang Road, Daqing City 163001, Heilong Jiang Province, China

**Keywords:** Carnitine, L-carnitine, Dose, Dosage, Dosing, Cardiovascular, Myocardial infarction, MI

## Abstract

**Background:**

L-carnitine supplementation has been associated with a significant reduction in all-cause mortality, ventricular arrhythmia, and angina in the setting of acute myocardial infarction (MI). However, on account of strict homeostatic regulation of plasma L-carnitine concentrations, higher doses of L-carnitine supplementation may not provide additional therapeutic benefits. This study aims to evaluate the effects of various oral maintenance dosages of L-carnitine on all-cause mortality and cardiovascular morbidities in the setting of acute MI.

**Methods:**

After a systematic review of several major electronic databases (PubMed, EMBASE, and the Cochrane Library) up to November 2013, a meta-analysis of five controlled trials (n = 3108) was conducted to determine the effects of L-carnitine on all-cause mortality and cardiovascular morbidities in the setting of acute MI.

**Results:**

The interaction test yielded no significant differences between the effects of the four daily oral maintenance dosages of L-carnitine (i.e., 2 g, 3 g, 4 g, and 6 g) on all-cause mortality (risk ratio [RR] = 0.77, 95% CI [0.57-1.03], *P* = 0.08) with a statistically insignificant trend favoring the 3 g dose (RR = 0.48) over the lower 2 g dose (RR = 0.62), which was favored over the higher 4 g and 6 g doses (RR = 0.78, 0.78). There was no significant differences between the effects of the daily oral maintenance dosages of 2 g and 6 g on heart failure (RR = 0.53, 95% CI [0.25-1.13], *P* = 0.10), unstable angina (RR = 0.90, 95% CI [0.51-1.58], *P* = 0.71), or myocardial reinfarction (RR = 0.74, 95% CI [0.30-1.80], *P* = 0.50).

**Conclusions:**

There appears to be no significant marginal benefit in terms of all-cause mortality, heart failure, unstable angina, or myocardial reinfarction in the setting of acute MI for oral L-carnitine maintenance doses of greater or less than 3 g per day.

## Background

Cardiovasular disease (CVD) is the world's leading cause of death, far outstripping infectious disease mortality from malaria, HIV/AIDS, and tuberculosis [1]. In the U.S., CVD is the leading cause of death, constitutes 17% of healthcare costs, and is estimated to increase in prevalence by ~10% and direct costs by almost three-fold over the next 20 years under current CVD prevention and treatment regimes [2]. However, secondary preventative interventions such as antiplatelet drugs, β-blockers, angiotensin-converting-enzyme (ACE) inhibitors, angiotensin-receptor blockers (ARBs), and HMG-CoA reductase inhibitors (statins) have been shown to significantly reduce CVD risk [3]. Therefore, a greater focus on improving the secondary prevention of CVD should ameliorate these dire projections.

One promising alternative therapy for the secondary prevention of CVD is L-carnitine, the biologically active stereoisomer of dietary carnitine (β-hydroxy-γ-N-trimethylaminobutyric acid) [4]. Cardiac muscle cells cannot synthesize L-carnitine *de novo* and must acquire L-carnitine exogenously via the arnitine/organic cation transporter 2 (OCTN2). Cardiac mitochondria use the carnitine-acylcarnitine carrier (CAC) to import fatty acyl moieties for β-oxidation, the primary energy source in heart muscle [4]. Thus, deficiencies in L-carnitine or its transporter CAC have particularly adverse effects on cardiomyocytes, resulting in cardiomyopathy, cardiac arrhythmia, cardiac insufficiency, and heart failure [4].

As exogenous L-carnitine aids in resumption of normal oxidative metabolism and restoration of myocardial energy reserves, L-carnitine supplementation has been shown to have favorable effects in CVD patients [4]. In chronic heart disease patients, L-carnitine administration over 12 months has been shown to attenuate left ventricular dilatation and prevent ventricular remodeling while reducing incidence of chronic heart failure and death. The protective effects of L-carnitine supplementation also extend to acute myocardial infarction (MI); following acute MI, prompt L-carnitine administration and subsequent oral maintenance therapy has been shown to attenuate progressive left ventricular dilatation and appears to reduce myocardial injury through improving carbohydrate metabolism and reducing the toxicity of high free fatty acid levels [5,6]. A recent systematic review and meta-analysis of 13 controlled trials by DiNicolantonio et al. found that L-carnitine supplementation was associated with a significant reduction in all-cause mortality, ventricular arrhythmia, and angina in the setting of acute MI [7].

However, the DiNicolantonio's study did not examine the effect of varying L-carnitine dosing on all-cause mortality or adverse cardiovasular outcomes. Following ingestion of a dietary L-carnitine, the rate of L-carnitine excretion increases rapidly because absolute reabsorption – which is based on the number of the OCTN2 transporters in the distal tubules of the kidney – does not change [8]. As L-carnitine levels do not significantly influence OCTN2 expression, renal filtration maintains a narrow plasma concentration range of 40–60 μmol/l with plasma L-carnitine above this threshold being eliminated via urine [8].

Therefore, on account of this strict homeostatic regulation of plasma L-carnitine concentrations, high doses of L-carnitine supplementation may not provide additional therapeutic benefits. In this systematic review and meta-analysis, we aim to determine the effects of various oral maintenance dosages of L-carnitine on all-cause mortality and cardiovascular morbidities in the setting of acute MI.

## Methods

### Ethics statement

The Ethics Committee (IRB) of Oilfield General Hospital (Daqing City, Heilong Jiang Province, China) recognizes that this systematic review and meta-analysis of de-identified, publicly available data does not constitute “human subjects research” as defined by relevant national regulations, and therefore does not require Ethics Committee review.

### Search strategy

A systematic review of the available literature was performed according to the PRISMA (preferred reporting items for systematic reviews and meta-analyses) guidelines for the conduct of systematic reviews of intervention studies [9]. Relevant randomized controlled trials (RCTs) were identified from systematic searches of several major electronic databases (PubMed, EMBASE, and the Cochrane Library) up to November 2013 with the following search strategy: (“L-carnitine” OR “carnitine”) AND “myocardial infarction”. English language and human study restrictions were imposed in all searches. Additional relevant articles were obtained through scanning reference lists of articles identified in the initial searches.

### Inclusion criteria

Studies were selected for inclusion on the basis of the following criteria: comparative trials of adults (> = 18 years old) receiving oral L-carnitine compared with placebo or control, with outcomes of all-cause mortality and adverse cardiovascular events including unstable angina, myocardial reinfarction, heart failure, and ventricular arrythmia. We excluded studies that did not report mortality or morbidity outcomes.

### Study selection and data extraction

The titles and abstracts of studies identified by the search strategy were independently screened by two reviewers, and irrelevant studies were excluded. The full texts were obtained from all articles meeting the inclusion criteria. Then, articles were scanned and the data from these studies was extracted, including the number of patients per arm, L-carnitine dosing (including initial loading dosing and daily oral maintenance dosing), acute MI (AMI) index event type, follow-up duration, and outcomes (i.e., all-cause mortality, adverse cardiovascular events including unstable angina, acute MI, heart failure, and ventricular arrythmia). Data extraction was performed by two independent reviewers.

### Quality assessment

Quality assessment was based on the following criteria – concealment of treatment allocation; similarity of both groups at baseline regarding prognostic factors and medication use; blinding of outcome assessors, care providers, and patients; completeness of follow-up; and intention-to-treat analysis – and quantified using the Jadad score [10]. Quality assessment was undertaken by two independent reviewers. Risk of bias was assessed using Cochrane Collaboration criteria specifically evaluating sequence generation of allocation; allocation concealment; blinding of participants, staff, and outcome assessors; incomplete outcome data; selective outcome reporting; and other sources of bias. Trials with high or unclear risk of bias in the first three criteria were deemed ‘high risk’.

### Statistical analysis

The meta-analysis of comparable data was performed using Review Manager 5.0.2 (the Nordic Cochrane Centre, the Cochrane Collaboration, 2008). For continuous outcomes, the results were expressed as the mean difference with a 95% confidence interval (CI). The degree of heterogeneity across the results of different studies was quantitatively assessed by the I^2^ statistic, with I^2^ < 30% indicating low heterogeneity, I^2^ = 30-50% indicating moderate heterogeneity, and I^2^ > 50% indicating substantial heterogeneity [11]. In the event of no conspicuous heterogeneity, a fixed-effects model was used. If heterogeneity was detected, a random-effects model was used. A *P* < 0.05 was deemed to be statistically significant for all analyses.

## Results

### Study selection and characteristics

**Figure 1:**
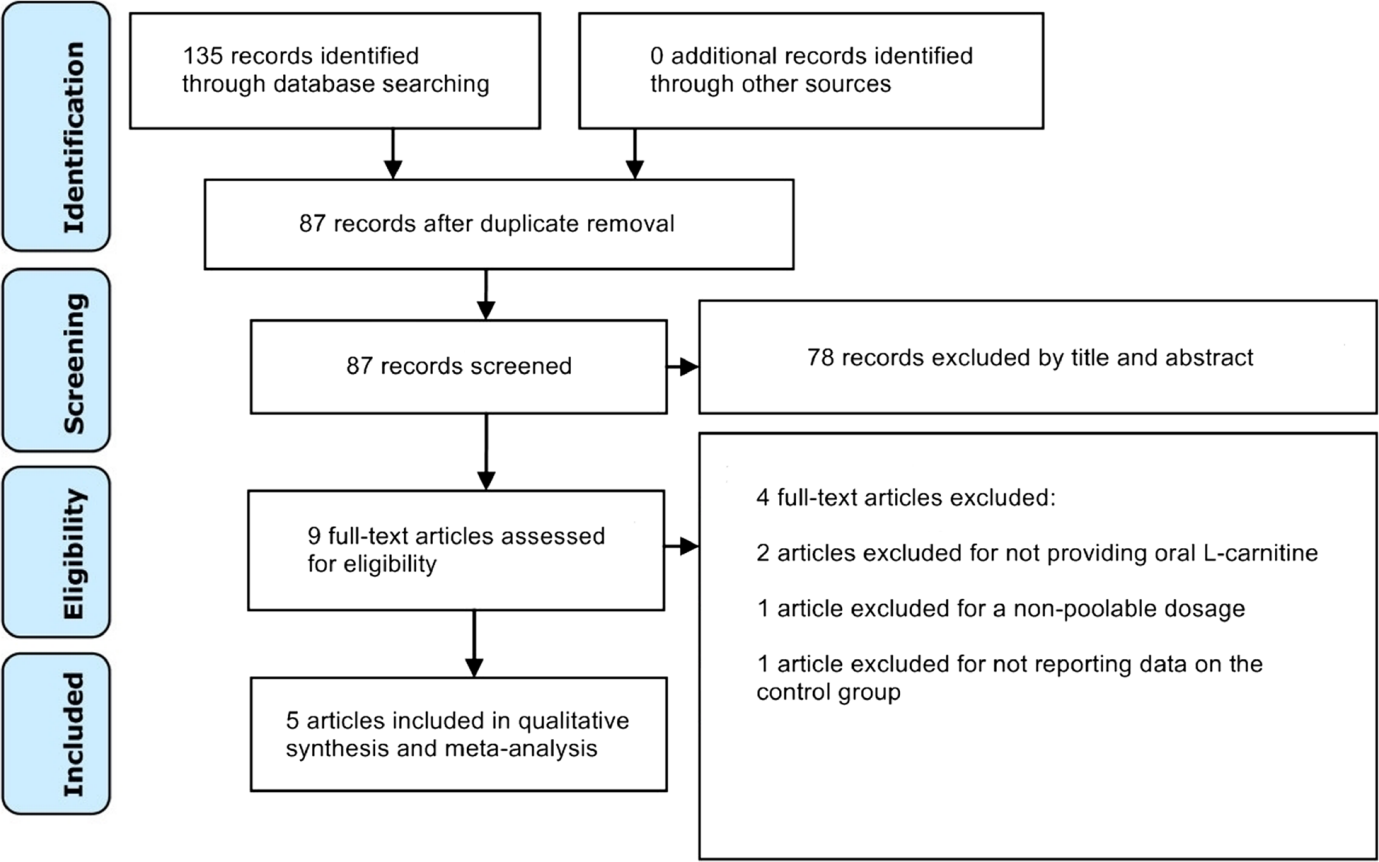
Flow diagram of literature search.

The literature search yielded 135 records (Figure 1). After screening titles and abstracts, only nine studies were eligible for full-text review, of which five met all inclusion criteria [12-16]. Table 1 summarizes the characteristics of the included studies including their risk of bias. All included trials were comparison trials of oral L-carnitine against placebo or control in the setting of acute MI. All background medications and baseline characteristics were statistically similar between the comparison groups in each trial.

**Table 1:** Characteristics of included studies

**Study**	**Sample size (n)**		**AMI index event type**	**Loading dose of L-carnitine**	**Oral maintenance dose of L-carnitine**	**Outcomes**	**Risk of Bias**^**†**^
	**L-carnitine treated**	**Control**					
Davini 1992	81	79	AMI	None	4 g/day	Death	◘◘◘
Iliceto 1995	233	239	AMI	9 g/day × 5 days (intravenous)	6 g/day	Death, bypass surgery, heart failure, early postinfarction angina, ventricular arrhythmia	◘◘○
Singh 1996	51	50	100% STEMI	None	2 g/day	Death, heart failure, unstable angina, reinfarction	◘◘○
Iyer 1999	23	23	Anterior AMI	6 g/day × 7 days (oral)	3 g/day	Death	◘◘◘
Tarantini 2006	1168	1161	100% STEMI	9 g/day × 5 days (intravenous)	4 g/day	Death	○○○

^†^Risk of bias assessment based on (i) sequence generation of allocation, (ii) allocation concealment, and (iii) blinding. The symbol ○ represents low bias risk, ● represents high bias risk, and ◘ represents unclear bias risk.

*Abbreviations*: *AMI* acute myocardial infarction, *STEMI* ST segment elevation myocardial infarction.

### Quality assessment

Table 2 summarizes the quality indicators of the included trials. All five studies scored well on the methodological quality indicators.

**Table 2:** Quality assessment of included studies

**Study**	**Jadad score**	**Allocation concealment**	**Similarity in baseline characteristics**	**Eligibility criteria**	**Blinding**			**Completeness of follow-up**	**Intention- to-Treat Analysis**
					**Assessor**	**Provider**	**Patient**		
Davini 1992	1	No	No	Yes	No	No	No	Yes	No
Iliceto 1995	4	Yes	Yes	Yes	Yes	Yes	Yes	Yes	No
Singh 1996	3	Yes	No	Yes	N/p	Yes	Yes	Yes	No
Iyer 1999	5	Yes	Yes	Yes	Yes	Yes	Yes	No	No
Tarantini 2006	3	Yes	Yes	Yes	N/p	Yes	Yes	Yes	Yes

N/p = Not provided.

### Study outcomes

**Figure 2:**
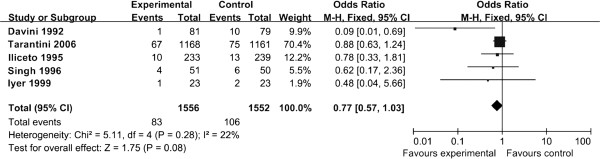
Forest plot of risk ratios for all-cause mortality.

All five trials (n = 3108) reported on all-cause mortality (Figure 2). The interaction test yielded no significant differences between the effects of the four daily maintenance dosages of L-carnitine (i.e., 2 g, 3 g, 4 g, and 6 g) on all-cause mortality (RR = 0.77, 95% CI [0.57-1.03], *P* = 0.08) (Figure 2). Analysis of the all-cause mortality risk ratios for each dosage yielded a statistically insignificant trend favoring the 3 g dose (RR = 0.48) over the lower 2 g dose (RR = 0.62), which was favored over the higher 4 g and 6 g doses (RR = 0.78, 0.78). There was low heterogeneity between trials for all-cause mortality (I^2^ = 22%).

**Figure 3:**
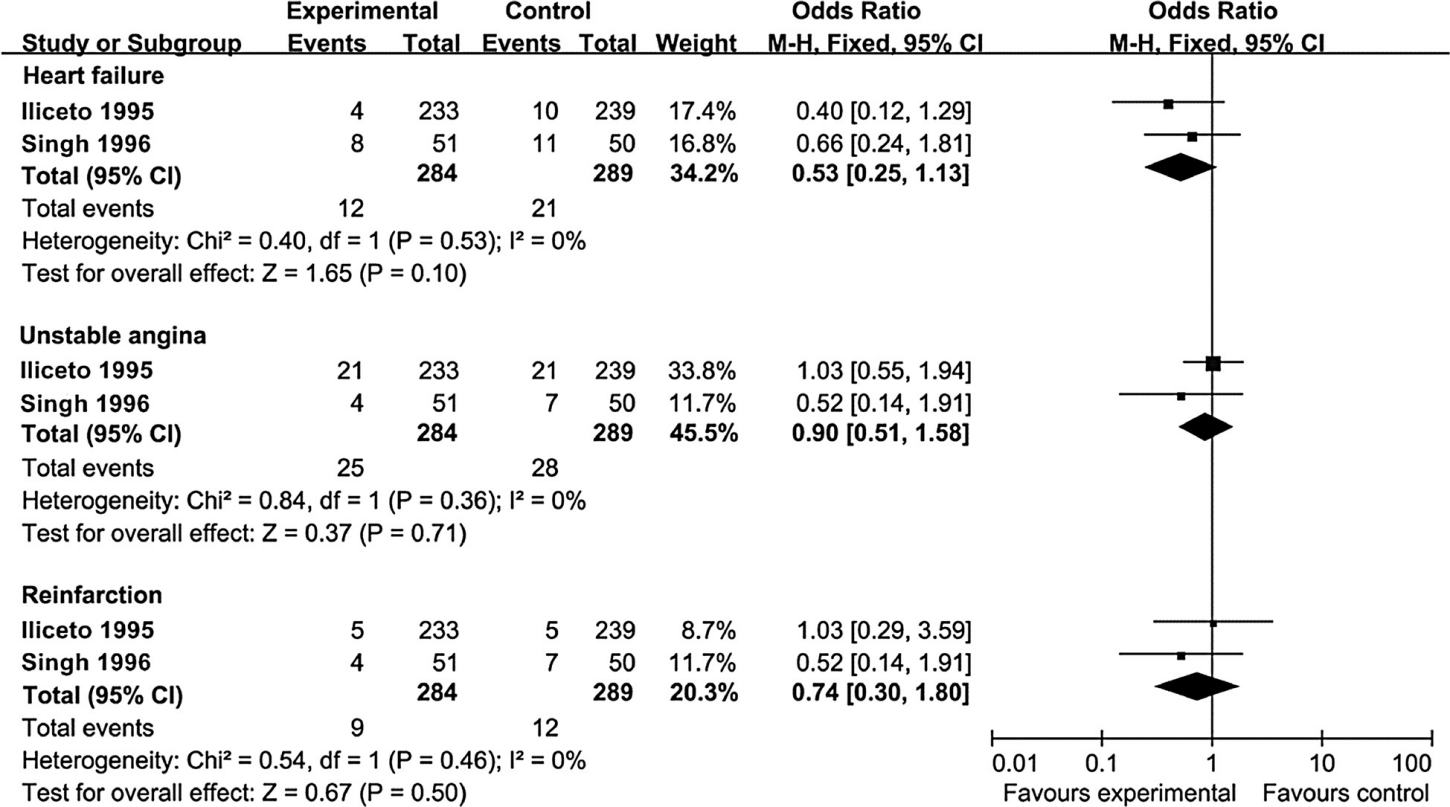
Forest plot of risk ratios for heart failure, unstable angina, and myocardial reinfarction.

Two trials, Iliceto 1995 (n = 472) and Singh 1996 (n = 101), reported on outcomes of heart failure, unstable angina, and myocardial reinfarction (Figure 3). There was no significant differences between the effects of the daily oral maintenance dosages of 2 g and 6 g on heart failure (RR = 0.53, 95% CI [0.25-1.13], *P* = 0.10), unstable angina (RR = 0.90, 95% CI [0.51-1.58], *P* = 0.71), or myocardial reinfarction (RR = 0.74, 95% CI [0.30-1.80], *P* = 0.50] (Figure 3). There was no heterogeneity between the two trials for all three outcomes (I^2^ = 0%).

## Discussion

Although L-carnitine supplementation has been associated with a significant reduction in all-cause mortality, ventricular arrhythmia, and angina in the setting of acute MI [7], this systematic review and meta-analysis of five controlled trials (n = 3108) found that there is no significant marginal benefit in terms of all-cause mortality, heart failure, unstable angina, or myocardial reinfarction for oral L-carnitine maintenance doses of greater than two grams per day. However, analysis of the all-cause mortality risk ratios for each dosage yielded a statistically insignificant trend favoring the 3 g dose over the lower 2 g dose, which was favored over the higher 4 g and 6 g doses. Although a statistically insignificant trend, this profile creates a bell-shaped curve with the 3 g dose as the optimal dosage in terms of all-cause mortality.

The human body's carnitine pool, consisting of free L-carnitine and its esters, is maintained by (i) absorption of L-carnitine from dietary sources, (ii) endogenous biosynthesis from two essential amino acids (lysine and methionine) in the kidney, liver and brain, and (iii) extensive renal tubular reabsorption (98-99%) from glomerular filtrate [17]. The absorption of oral L-carnitine occurs via both passive diffusion and carrier-mediated transport, which ensures high tissue-to-plasma concentration ratios in tissues that depend critically on fatty acid oxidation. The bioavailability from conventional oral supplements (one to six grams) ranges from a mere 5-18% [17]. One reason for this low bioavailability is a significant proportion of L-carnitine supplementation is metabolized by microbiota prior to absorption [18,19]. Human microbiota are responsible for converting L-carnitine and other dietary quaternary amines (e.g., choline, glycine betaine, and phosphatidylcholine) to trimethylamine (TMA), which are subsequently oxidized by host hepatic flavin monooxygenases to trimethylamine N-oxide (TMAO), a molecule that promotes atherogenesis through its interaction with macrophages and lipid metabolism [20,21]. Thus, gut microbiota may not only reduce L-carnitine bioavilability but also promote TMAO-induced atherosclerotic risk. Interestingly, this limited bioavailability of oral L-carnitine supplements may have been a motivating rationale for the high dosing of oral L-carnitine supplementation (i.e., 2–6 g daily). Although gut microbiome profiles and L-carnitine bioavailability were not reported in the studies included in this meta-analysis, future studies should measure these variables when assessing the efficacy and dosing of L-carnitine in CVD patients, as certain gut microbiota species (e.g., *Gammaproteobacteria*, *Betaproteobacteria*, and *Firmicutes*, including *Acinetobacter* species) [22] can display a particularly adverse influence on the bioavailability of L-carnitine supplementation as well as atherosclerotic risk through TMA production.

As the vast majority (>95%) of the human body's carnitine pool is located in skeletal muscle, the dynamics of skeletal muscle carnitine may influence the metabolism of L-carnitine supplementation. In humans, increased plasma concentrations have not been conclusively associated with an increase in the skeletal muscle carnitine pool [23]. This phenomenon may be due to saturation of L-carnitine transport into skeletal muscle at physiological L-carnitine plasma concentrations (40–60 μmol/l) and/or by the significantly higher carnitine concentration in skeletal muscle relative to plasma, rendering passive transport impossible [24]. However, other human studies have found opposing results. Studies providing month-long oral supplementation of 2 g of L-carnitine per day to long-distance runners (which consume large amounts of carbohydrates for training) showed a ~10% increase in skeletal muscle carnitine content [25,26]. Moreover, recent studies by Stephens and Wall providing human subjects with twice daily 1.36 g L-carnitine in combination with a beverage containing 80 g of carbohydrate reported a 20% increase in skeletal muscle carnitine content over a 12-week period and a 30% increase in skeletal muscle carnitine content over a 24-week period [27,28]. These discrepancies may be explained by the hypothesis that L-carnitine supplementation accompanied by high amounts of carbohydrate intake increases skeletal muscle OCTN2 expression through an insulin-mediated mechanism, thereby increasing skeletal muscle carnitine content [28,29]. Although skeletal muscle carnitine content and carbohydrate intake were not reported in the studies included in this meta-analysis, future studies should measure these variables when assessing the efficacy and dosing of L-carnitine in CVD patients, as these factors may influence the *in vivo* metabolism of L-carnitine supplementation.

Although previous studies have shown L-carnitine to have cardioprotective effects [4-7], recent studies have also shown that derivatives of L-carnitine may have adverse consequences on cardiovacular health. As discussed earlier, Koeth et al.'s study on gut microbiota-induced TMAO in stable patients undergoing cardiac evaluation showed significant dose-dependent associations between plasma L-carnitine levels and risks of coronary artery disease, peripheral artery disease, and overall CVD after correction for common CVD risk factors [18]. Moreover, the same study demonstrated that elevated fasting plasma L-carnitine levels are an independent predictor of major adverse cardiac events after correction for common CVD risk factors [18]. In another recent clinical study, higher plasma levels of the L-carnitine derivates acetylcarnitine and palmitoylcarnitine have been associated with higher degrees of heart failure, and higher plasma levels of palmitoylcarnitine have been associated with higher rates of all-cause mortality and heart transplantation [30]. As these recent studies show that higher oral doses of L-carnitine can promote atherogenesis and CVD risk, these findings also support our contention of limiting the oral maintenance dosing of L-carnitine to 3 g per day in the setting of acute MI and call for further investigation on the long-term risks of chronic L-carnitine supplementation in CVD patients.

Several limitations to this study should be noted here. First, three of the five trials included in the meta-analysis possessed a relatively small number of patients (n < 200) – the two large trials, Iliceto 1995 (n = 472) and Tarantini 2006 (n = 2329), contributed 87% of the mortality events. However, we found low heterogeneity between the five trials in all-cause mortality (I^2^ = 22%) (Figure 2) and detected no heterogeneity between the two trials reporting heart failure (I^2^ = 0%), unstable angina (I^2^ = 0%), and myocardial reinfarction (I^2^ = 0%) (Figure 3). Second, due to lack of reported data, we could not analyze L-carnitine dosing in the secondary prevention of ventricular arrythmia in the setting of acute MI. Third, all included studies were conducted prior to 2006; as standard treatment regimens for acute MI patients have changed substantially since then (e.g., revascularization with dual antiplatelet therapy, HMG CoA reductase inhibitors), the potential benefits of L-carnitine will need to be reassessed in the context of current treatment regimens that may affect the pharmacokinetics of L-carnitine. Fourth, only the effects of the four daily oral maintenance dosages (2 g, 3 g, 4 g, and 6 g) were analyzed here. Therefore, we could not ascertain whether daily oral maintenance doses under 2 g or above 6 g are equally effective, nor did we examine the effects of different initial loading administrations. Fifth, due to lack of reported data, we could not analyze the dietary patterns, medication usage, gut microbiome profiles, L-carnitine bioavailability, or skeletal muscle carnitine content of the patients included in this meta-analysis, which may have had differential effects on the metabolism of L-carnitine. Sixth, the follow-up periods of the included trials were relatively short-term (one to twelve months); therefore, interpretation of these findings are restricted to shorter-term outcomes. Seventh, these results should not be applied to patients with primary carnitine deficiency (typically arising from genetic alterations in renal handling or muscle transport of L-carnitine) or secondary carnitine deficiency (typically arising from impaired renal tubular resorption from drug toxicity or hemodialysis). Other studies have analyzed the pharmacokinetics and provided dosing recommendations regarding L-carnitine in these patients [31,32].

## Conclusions

There appears to be no significant marginal benefit in terms of all-cause mortality, heart failure, unstable angina, or myocardial reinfarction in the setting of acute MI for oral L-carnitine maintenance doses of greater or less than 3 g per day.

## Competing interests

The authors declare that they have no competing interests.

## Authors’ contributions

RS conceived and designed the experiments. RS and ZS collected the data. ZS and HL analyzed the data. RS and HL drafted and critically revised the manuscript. RS supervised the study. All authors read and approved the final manuscript.

## Pre-publication history

The pre-publication history for this paper can be accessed here:

http://www.biomedcentral.com/1471-2261/14/88/prepub
